# Regulation of Olfactory Associative Memory by the Circadian Clock Output Signal Pigment-Dispersing Factor (PDF)

**DOI:** 10.1523/JNEUROSCI.0782-20.2020

**Published:** 2020-11-18

**Authors:** Johanna G. Flyer-Adams, Emmanuel J. Rivera-Rodriguez, Junwei Yu, Jacob D. Mardovin, Martha L. Reed, Leslie C. Griffith

**Affiliations:** Department of Biology, Volen National Center for Complex Systems, Brandeis University, Waltham, Massachusetts 02454-9110

**Keywords:** clock, *Drosophila*, memory, mushroom body, neuropeptide

## Abstract

Dissociation between the output of the circadian clock and external environmental cues is a major cause of human cognitive dysfunction. While the effects of ablation of the molecular clock on memory have been studied in many systems, little has been done to test the role of specific clock circuit output signals. To address this gap, we examined the effects of mutations of *Pigment-dispersing factor* (*Pdf*) and its receptor, *Pdfr*, on associative memory in male and female *Drosophila*. Loss of PDF signaling significantly decreases the ability to form associative memory. Appetitive short-term memory (STM), which in wild-type (WT) is time-of-day (TOD) independent, is decreased across the day by mutation of *Pdf* or *Pdfr*, but more substantially in the morning than in the evening. This defect is because of PDFR expression in adult neurons outside the core clock circuit and the mushroom body (MB) Kenyon cells (KCs). The acquisition of a TOD difference in mutants implies the existence of multiple oscillators that act to normalize memory formation across the day for appetitive processes. Interestingly, aversive STM requires PDF but not PDFR, suggesting that there are valence-specific pathways downstream of PDF that regulate memory formation. These data argue that the circadian clock uses circuit-specific and molecularly diverse output pathways to enhance the ability of animals to optimize responses to changing conditions.

**SIGNIFICANCE STATEMENT** From humans to invertebrates, cognitive processes are influenced by organisms' internal circadian clocks, the pace of which is linked to the solar cycle. Disruption of this link is increasingly common (e.g., jetlag, social jetlag disorders) and causes cognitive impairments that are costly and long lasting. A detailed understanding of how the internal clock regulates cognition is critical for the development of therapeutic methods. Here, we show for the first time that olfactory associative memory in *Drosophila* requires signaling by Pigment-dispersing factor (PDF), a neuromodulatory signaling peptide produced only by circadian clock circuit neurons. We also find a novel role for the clock circuit in stabilizing appetitive sucrose/odor memory across the day.

## Introduction

Cognition is influenced by the circadian clock. Within individual cells, time is kept by a molecular clock; the coordination of time across tissues in the organism is performed by the 'core clock', a small population of individually cycling neurons bound together to form a coherent circuit. The outputs of this circuit generate rhythms of physiology and behavior that oscillate with a period aligned to the solar cycle. Circadian peaks and troughs in learning and memory have been documented in humans as well as rodent and invertebrate models (Gerstner and Yin, 2010; Wright et al., 2012) such that misalignments between an organism's cycling internal clock and external conditions (jetlag, social jetlag, and shift work disorders) impair cognition/memory across phyla (Wittmann et al., 2006; Kott et al., 2012; Weingarten and Collop, 2013; Wright et al., 2013). Irreversible neurodegenerative diseases that affect cognition such as Parkinson's and Alzheimer's exhibit comorbid circadian dysfunction (Videnovic et al., 2014). Even natural aging degrades the fidelity of the body's clock in a manner that has been linked to cognitive decline (Reinhart and Nguyen, 2019). Thus, a complete understanding of how the body's clock regulates cognition could generate new therapeutic approaches.

To date, the clock-memory link has largely been investigated by breaking the transcriptional feedback loop providing intracellular timekeeping in cells of the clock circuit. Eliminating cycling with altered light conditions affects novel object recognition in rodents (Ruby et al., 2013) and time-of-day (TOD)-dependent associative memory in *Drosophila* (Lyons and Roman, 2009; Le Glou et al., 2012; Chouhan et al., 2015). Clock gene mutations such as *Per1*, *Per2*, and *Bmal1* generate impairments in contextual fear and spatial memory tasks in mice and are involved in all phases of memory processing (Wang et al., 2009; Rawashdeh et al., 2014; Wardlaw et al., 2014; Snider and Obrietan, 2018). In *Drosophila*, mutations of the clock genes *period* and *clock* impair TOD memory and can disrupt memory generally (Sakai et al., 2004; Lyons and Roman, 2009; Le Glou et al., 2012; Fropf et al., 2014, 2018; Chouhan et al., 2015). But studies using molecular clock mutants do not fully replicate conditions that exist in common human clock-related cognitive disorders; in most, a functional molecular clock is present but outputs are misaligned with environmental cues. To fully understand the role of clock circuit outputs on cognition, it is necessary to manipulate output pathways in the context of an intact molecular clock.

Environmentally-cued circadian rhythms of behavior and physiology are generated by the outputs of the core clock circuit which are organized by peptidergic signaling from a few pacemaker neurons (Aton et al., 2005). In *Drosophila*, this peptide is Pigment-dispersing factor (PDF), an 18-amino acid peptide produced in the adult brain solely by 16 ventrolateral clock neurons (LNvs; Renn et al., 1999; Park et al., 2000). PDF released from the LNvs signals within the core clock circuit through its only known receptor PDFR (Hyun et al., 2005; Lear et al., 2005; Mertens et al., 2005; Shafer et al., 2008) to coordinate the activity of the ∼150 core clock neurons (Peng et al., 2003; Lin et al., 2004; Stoleru et al., 2005; Yoshii et al., 2009; Liang et al., 2016). Core clock circuit output directs rhythmic aspects of physiology such as locomotor activity and sleep, and PDF also functions in these output pathways. In this way, the PDF/PDFR signaling pathway both maintains free-running circadian activity and promotes output behaviors.

Here, we investigate the involvement of PDF signaling in *Drosophila* memory, examining the role of clock output on associative olfactory memory for the first time in the context of a functional molecular clock. We find a novel adult-specific requirement for PDF/PDFR signaling in memory that is distinct from its role in synchronizing the clock circuit. We also provide evidence for the existence of a second PDF receptor that allows valence-specific regulation of associative olfactory memory by PDF.

## Materials and Methods

### 

#### Fly stocks and husbandry

**Figure 1.**
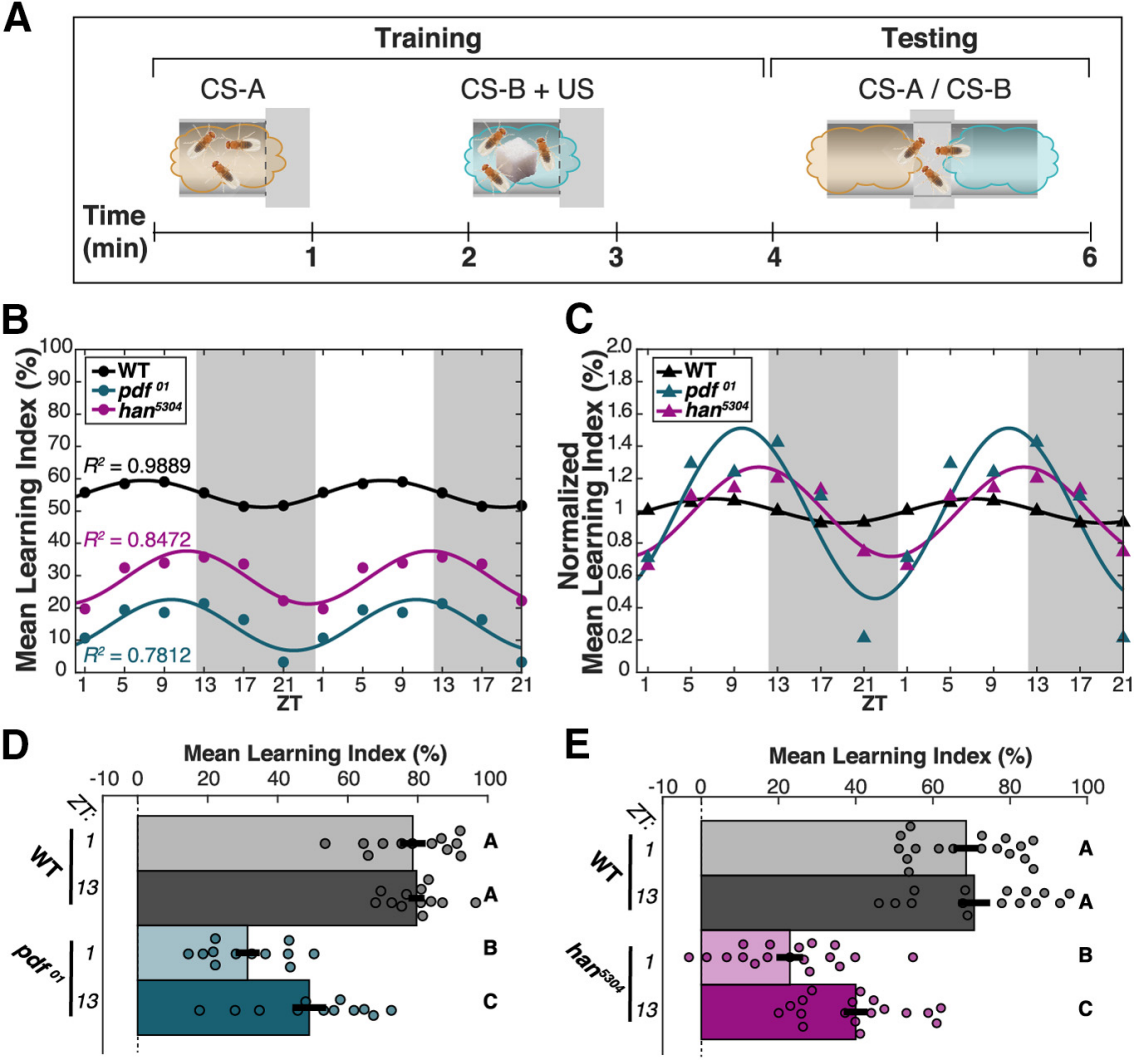
The core clock supports appetitive STM throughout the circadian cycle via a PDF signaling pathway. Appetitive olfactory 2-min STM of WT and PDF pathway mutants. ***A***, A cartoon protocol of appetitive olfactory 2-min STM training and testing shows 1-min pairings of two neutral CSs (odor) with and without US (sucrose) interspersed with 1 min rest and followed directly by a 2-min CS choice test. ***B***, ***C***, LI scores for STM tested every 4 h through the 24-h cycle and double-plotted, with white and gray background indicating lights on/off of entrainment phase, Zeitgeber time (ZT) as indicated. Each genotype was fit with one-term Fourier curve, *R*^2^ values shown in ***A***. ***B***, Mean LI (circles; *n* = 8 for each time point) and ***C*** normalized mean LI (triangles; mean subtracted, then divided by SD). ***D***, ***E***, STM of *pdf^01^* and *han^5304^* mutants compared with WT at ZT1 and ZT13. Mean LI scores are shown ± SEM, with individual datapoints (circles). Letter categorization indicates groups of statistical equivalence (*p* > 0.05) or difference found by two-way ANOVA with interactions, Bonferroni *post hoc* comparisons (all significant comparisons, ***D***: *p* < 0.01, ***E***: *p* < 0.005).

All experimental flies were collected directly after eclosion and maintained in incubators at 25°C with a 12/12 h light/dark (12/12 LD) cycle. Flies were reared and housed on cornmeal dextrose food with yeast except those used for GeneSwitch experiments. For Figure 1, 24hr memory, and aversive memory experiments, mixed males and females were used; for cell-specific PDFR expression memory experiments and all activity experiments, males were used; for imaging experiments, females were used. Fly strains used include: *Canton-S* wild-type (WT), ;;*pdf^01^* (BDSC#26654; backcrossed 6× into WT), *han^5304^*;; (BDSC#33068; backcrossed 6× into WT), ;;*nsyb-GAL4/TM6B* (Goodwin et al., 2018), *han^5304^;;UAS-pdfr-myc13* (gift of Paul Taghert), *;;elav-GS-GAL4* (Osterwalder et al., 2001), *VT030559-GAL4* (VDRC ID# v206077), *pdfR-2A-LexA;;* and *;;pdf^attP^* (Deng et al., 2019), *w^-^,UAS-mCD8-IVS-RFP,LexAop-mCD8-IVS-GFP;;* (generated from BDSC#61681), ;*pdf-GAL4;* (Park et al., 2000), *UAS-P2X_2_* (Lima and Miesenböck, 2005), *MB-LexA* (Pitman et al., 2011), *clk4.1-GAL4* (Zhang et al., 2010), ;*LexAop-EPAC;* (BDSC#76 031), ;*pdfR(10 kb)-GAL4;* (Parisky et al., 2016), ;*clk856-GAL4;* (gift from M. Rosbash).

#### Temporal panneuronal expression of pdfr (elav-GS-GAL4, adult only)

GeneSwitch experimental flies (*han^5304^;;elav-GS-GAL4/UAS-Pdfr*) were housed after eclosion on normal yeast food containing 0.2 g/L RU486 (Sigma, catalog #8046) diluted into 100% ethanol; control flies were housed after eclosion on normal food containing equal volume 100% ethanol. Total exposure time was 6–12 d. Flies were starved for appetitive learning assays on nutrient-free agarose ± RU486 or ethanol control.

#### Learning assays

Appetitive and aversive associative olfactory memory assays were performed in an environmental room in red light at 25°C with 65% ambient humidity. Flies were between 4–14 d old [short-term memory (STM)] or 4–10 d old [long-term memory (LTM)] and given at least 10-min acclimation to the environmental room before training or testing. Data for each experiment was pooled from at least three independent experimental days.

Appetitive learning assays were performed as previously described (Liu et al., 2012) using a modification of the methods pioneered by the Quinn lab (Tempel et al., 1983; Tully and Quinn, 1985). Briefly, flies were starved to 10% mortality. Filter papers were prepared blank or with 2 M sucrose as null or unconditioned stimulus (US), and 10% MCH and OCT prepared as conditioned stimulus (CS) odors. As schematized in Figure 1*A*, 50–100 starved flies were loaded into a vial and exposed to a one trial training of sequential CS_A_-null and CS_B_-US pairings of 1-min (STM) or 2-min (LTM) duration. Flies were then either tested for CS preference (2-min STM) or placed into fresh food vials for 4 h and then starved for 20 h before CS preference testing (24-h LTM). Testing involved a 2-min sequential exposure to CS odors, after which flies choosing either odor were counted. A preference index (PI) was calculated for each trial as [(# of flies in CS_A_) – (# of flies in CS_B_)]/[(# of flies in CS_A_) + (# of flies in CS_B_)]. This PI was averaged with the PI of a temporally-paired CS reciprocal trial to generate the final learning index (LI), calculated as a percentage. In this way, each datapoint shown for these experiments represents 100–200 flies and controls for odor bias. To confirm mutant detection of unpaired stimuli, 2-min preference tests were performed (Table 1) during which flies chose between a stimulus vial (containing either 10% OCT, 10% MCH, 2 m sucrose, or 24 spaced 1-s 90-V shocks) and a neutral vial (PI calculated as above.) Mutant flies showed preferences equivalent to WT for OCT (two-way ANOVA: *p*_gen_ = 0.086, *p*_ZT_ = 0.52, *p*_genxZT_ = 0.90), sucrose (two-way ANOVA: *p*_gen_ = 0.69, *p*_ZT_ = 0.43, *p*_genxZT_ = 0.93), and shock (one-way ANOVA: *p*_WT,pdf01_ = 0.736, *p*_WT,han5304_ = 0.141, *p*_pdf01,han5304_ = 0.027), while *han^5304^* flies showed altered MCH preference compared with WT and *pdf^01^* mutants (two-way ANOVA: *p*_gen_ = 0.0015, *p*_ZT_ = 0.47, *p*_genxZT_ = 0.93; Bonferroni *post hoc* for effect of genotype: *p*_WT,pdf01_ = 0.39, *p*_WT,han5304_ = 0.0012, *p*_pdf01,han5304_ = 0.046).

**Table 1.** Mutant stimulus preference

		OCT	MCH	Sucrose	Shock
ZT1	*WT*	−8.99 ± 17.69	−11.44 ± 16.67	70.19 ± 7.73	−52.1 ± 4.9
	*pdf^01^*	−28.43 ± 6.88	3.56 ± 11.36	66.58 ± 10.99	−46.4 ± 7.3
	*han^5304^*	2.61 ± 10.65	42.54 ± 11.41	61.13 ± 9.43	−67.0 ± 3.5
ZT13	*WT*	−6.51 ± 12.51	−20.12 ± 14.98	72.13 ± 5.04	-
	*pdf^01^*	−16.20 ± 6.02	1.13 ± 15.23	74.90 ± 7.67	-
	*han^5304^*	6.81 ± 13.42	29.56 ± 11.19	67.79 ± 10.21	-

Starved mixed male and female flies were tested at ZT1 and ZT13 for innate (unpaired) odor and sucrose preference. Data reported as the mean ± SEM (odor and sucrose, *n* = 8 per group; shock, *n* = 16/group). Two-way ANOVA shows no significant differences of genotype, ZT, or interactions for OCT and sucrose preference (*p* > 0.05). Two-way ANOVA for MCH preference shows no significant differences of ZT or interactions (*p* > 0.05), shows effect of genotype (*p*_gen_< 0.005; Bonferroni *post hoc* comparison: *p*_WT_,_han5304_ and *p*_pdf01,han5304_ < 0.05, *p*_WT_,_pdf01_ = n.s.) shock preference one-way ANOVA shows effect of genotype (*p*_gen_ < 0.05; Bonferroni *post hoc* comparison: *p*_pdf01_,_han5304_ < 0.05, *p*_WT,pdf01_ and *p*_WT_,_han5304_ = n.s.)

Aversive learning STM assays were performed similarly to appetitive STM assays with the following modifications. Flies were not starved before training, and the US was provided by supplying 12 1-s 90-mV shocks during the 1-min CS-US pairing. For appetitive STM, flies were immediately tested for CS preference and a final LI calculated from paired reciprocal tests.

#### Activity assays and data extraction

Male flies were collected immediately after eclosion and individually loaded into sleep tubes and detectors as described previously (Haynes et al., 2015). Flies were entrained for 3 d to 12/12 LD in 25°C incubators, after which 3 d of 12/12 LD baseline activity was collected followed by release into free-running DD conditions for 9 d. During this time locomotor activity data were collected using the *Drosophila* Activity Monitor (DAM; TriKinetics) as previously described (Agosto et al., 2008). Subsequently, beam break counts were extracted using DAMFileScan (TriKinetics) and analyzed for sleep and activity using MATLAB SCAMP (v.2019, Chris Vecsey, Skidmore) as done previously (Haynes et al., 2015). DD analysis was performed on days 2–8 DD data, with percent of population rhythmic values calculated using cutoff criteria where a rhythmicity index (RI) below 0.2 was considered arrhythmic. LD data were calculated from an average of days 1–3 in LD. Custom MATLAB scripts were written to extract evening anticipation onset and evening peak values from 30-m binned LD activity data of individual subjects and are available on GitHub (https://github.com/Griffith-Lab). Evening anticipation onset was calculated as the max value of the three-bin rolling average of the first derivative for data between Zeitgeber time (ZT)6-ZT11.5 (to prevent artifact from lights-off startle effects). Evening peak was calculated as the max value between ZT6 and ZT12.

#### Imaging

Functional imaging was performed *ex vivo* in whole brains with imaging hardware, solutions and acquisition software as described previously (Haynes et al., 2015). Planar image acquisition in MB calyx and surrounding soma (via *MB-LexA*) or DN1 clock neuron soma (via *clk4.1-GAL4*) was performed at 2 Hz. For each brain, both an AHL control and drug (ATP, PDF, or FSK) experimental trial was performed, each including 30 s of baseline AHL perfusion followed by 120 s of drug or vehicle perfusion. Thus, these groups were statistically compared by paired *t* test. For Figure 3*E*, an additional unpaired *t* test was used to compare the experimental group (*pdf*>*P2X2,MB*>*EPAC* +ATP) to the empty driver control (*P2X2,MB*>*EPAC* +ATP) which received 30 s of baseline AHL followed by 120 s of ATP perfusion. Data were processed before analysis by custom MATLAB scripts which normalized each trace to its baseline and then fit and subtracted out any linear trends present in baseline because of bleaching (available on GitHub: https://github.com/Griffith-Lab). Expression pattern imaging was performed as follows: brains were fixed, stained, mounted and embedded according to Janelia FlyLight protocols (Dissection and Fixation 2% PFA, IHC-Double Label, DPX Mounting; https://www.janelia.org/project-team/flylight/protocols). Primary antibodies included mouse monoclonal anti-GFP (1:200; Roche Applied Science, catalog #11814460001), and rabbit polyclonal anti-Ds-red (1:200; Clontech, catalog #632496). Secondary antibodies included Cy2 goat anti-mouse (1:400; The Jackson Laboratory, catalog #115-225-166) and Alexa Fluor 594 donkey anti-rabbit (1:400; The Jackson Laboratory, catalog #711-585-152) as recommended in Janelia protocols. Samples were imaged on a Zeiss LSM 880 microscope using a Plan-Apochromat 25×/0.8 Imm Corr DIC M27 objective. Data presented in Figure 3*B*,*C*, Movie 1 were imaged using 561/595 and 488/515 channels with a slice depth of 0.65 µm and subsequently processed with Airyscan. Data presented in Figure 3*D*, Movie 2 were acquired with a slice depth of 0.75 µm using 458/550 and 633/698 channels. 3D rendering was performed on Zeiss Zen 2.3 software.

Movie 1. Putative PDFR expression is excluded from the MB KCs. Confocal image of MB calyx and KC soma, acquired with a slice depth of 0.65 µm, processed with Airyscan, and rendered as a fly through 5-fps video (posterior→anterior). MB calyx and KC soma are shown in magenta (fixed with dsRed antibody staining of *VT030559-GAL4*>*UAS-mCD8-IVS-RFP*); putative PDFR expression pattern is shown in green (fixed with GFP antibody staining of *pdfR-2A-LexA*>*LexAop-mCD8-IVS-GFP*). 10.1523/JNEUROSCI.0782-20.2020.video.1

Movie 2. Putative PDFR expression is excluded from the MB lobe neuropil. Confocal image of MB lobe neuropil, acquired with a slice depth of 0.75 µm, and rendered as a 3D rotation around the *x*-axis. MB lobes are shown in magenta (fixed with dsRed antibody staining of *VT030559-GAL4*>*UAS-mCD8-IVS-RFP*); putative PDFR expression is shown in green (fixed with GFP antibody staining of *pdfR-2A-LexA*>*LexAop-mCD8-IVS-GFP*). The MB β/β' lobe tips hug the PDFR^+^ EB projection rings without overlap. 10.1523/JNEUROSCI.0782-20.2020.video.2

#### Experimental design and statistical analysis

Information regarding experimental design for both behavior and imaging can be found in those sections within Materials and Methods. All statistical analyses used are detailed in Results and figure legends and were performed using MATLAB R2019a (MathWorks). As the Kolmogorov–Smirnov test for normality is known to be overly sensitive with small sample sizes, statistical approaches accommodating unequal variance or non-parametric data were used in cases (1) where maximum SD was equal or greater than three times' the minimum SD, or (2) there was a known ceiling/floor imposing non-normality. Within a figure, statistically similar groups (*p* > 0.05) are identified by the same letter assignment; statistically different groups (*p* < 0.05) can be identified by having differing letter group assignments. For all tests requiring *post hoc* comparisons (ANOVA, Kruskal–Wallis), the Bonferroni method was used. For any significant (*p* < 0.05) *post hoc* comparison *p* values of an analysis, the largest significant *p* value informed the selection of the closest standard *p* values ultimately reported in figure legends (i.e., *p* < 1E-4 in the case where the largest *p* value was 0.000045).

## Results

### WT appetitive olfactory STM is stable throughout the day

While it has previously been shown that there are TOD effects on some forms of olfactory memory in *Drosophila* (Lyons and Roman, 2009; Fropf et al., 2014, 2018; Chouhan et al., 2015), there has been no investigation of the role of the clock in appetitive olfactory STM. To address this, we first tested appetitive STM of *Canton-Special* WT flies entrained to a 12/12 LD cycle at six timepoints evenly spaced throughout the 24-h day (Fig. 1*B*, black). Grouped flies (100–200) were exposed to two neutral odors, one paired with sucrose and the other unpaired. Memory of this experience was assessed directly after training by allowing animals to choose between the two odors; WT flies prefer the odor previously paired with sucrose (Tempel et al., 1983). While a double-plot of the mean LI of each time point was best fit with a nonlinear one-term Fourier curve (*R*^2^ = 0.9889; linear fit *R*^2^ = 0.5400), there were no statistically significant TOD changes for WT flies (*n* = 8 per time point, two-way ANOVA, *p*_ZT_ = 0.0373; *post hoc* comparisons for WT×ZT, all *p* = 1.00). Thus, WT appetitive STM appears to be relatively stable throughout the 24-h circadian cycle.

### The magnitude and TOD-independence of appetitive STM requires PDF/PDFR signaling

To determine whether the circadian clock has a role in regulation of appetitive STM, we asked whether the major molecular output of the core clock, the neuropeptide PDF (Renn et al., 1999; Shafer and Yao, 2014) and its receptor PDFR (Hyun et al., 2005; Lear et al., 2005; Mertens et al., 2005; Choi et al., 2009), affected this behavior. Mutants lacking PDF (*pdf^01^*) or PDFR (*han^5304^*) were tested for appetitive STM alongside WT flies (Fig. 1*B*). Both *pdf^01^* and *han^5304^* showed a clear STM deficit compared with WT at all time points (two-way ANOVA: *p*_genotype_ = 6.43E-23; Bonferroni *post hoc* comparisons: *p*_WT,pdf_ = 3.62E-23, *p*_WT,han_ = 4.31E-12, *p*_pdf,han_ = 3.44E-05). All genotypes were responsive to odor, sugar and shock (see Materials and Methods; Table 1). Given the requirement found for both PDF and its receptor PDFR, we conclude that the PDF signaling pathway supports STM throughout the day.

These *pdf^01^* and *han^5304^* data were also well fit by nonlinear Fourier curves (*R*^2^ = 0.7812 and *R*^2^ = 0.8472, respectively; linear fit *R*^2^ = 0.0052 and *R*^2^ = 0.0987, respectively), but their apparent amplitudes of oscillation were larger than those of WT. This is made more obvious if the data are normalized to their means to allow direct comparison between genotypes (Fig. 1*C*). While this normalization dampens the amplitude of the WT oscillation, the *pdf^01^* and *han^5304^* curves show even larger excursions, implying that the PDF signaling pathway may in fact stabilize STM formation over the course of the day.

To test this, we assayed WT STM compared with *pdf^01^* or *han^5304^* at the TODs where we had previously observed largest deviations from the mean: dawn (ZT1) and dusk (ZT13; Fig. 1*D*,*E*). These experiments replicated the prior overall deficits seen in *pdf^01^* and *han^5304^* appetitive STM and confirmed that WT appetitive STM does not change with TOD [two-way ANOVA, *post hoc* comparisons: *p*_WT1,13_ = 1.00 (Fig. 1*D*); *p*_WT1,13_ = 1.00 (Fig. 1*E*)]. However, as predicted by our qualitative observations in Figure 1*B*,*C*, both *pdf^01^* and *han^5304^* had significantly lower learning scores at ZT1 relative to ZT13 [two-way ANOVA, *post hoc* comparisons: *p*_pdf1,13_ = 0.008 (Fig. 1*D*); *p*_han1,13_ = 0.003 (Fig. 1*E*)]. The loss of PDF signaling through PDFR therefore uncovers strong TOD effects on the ability to learn. We conclude that PDF and its receptor PDFR are generally necessary for STM but also have a critical role in ensuring that animals can learn equally well at all TODs.

### Appetitive STM requires PDF signaling in adult neurons

**Figure 2.**
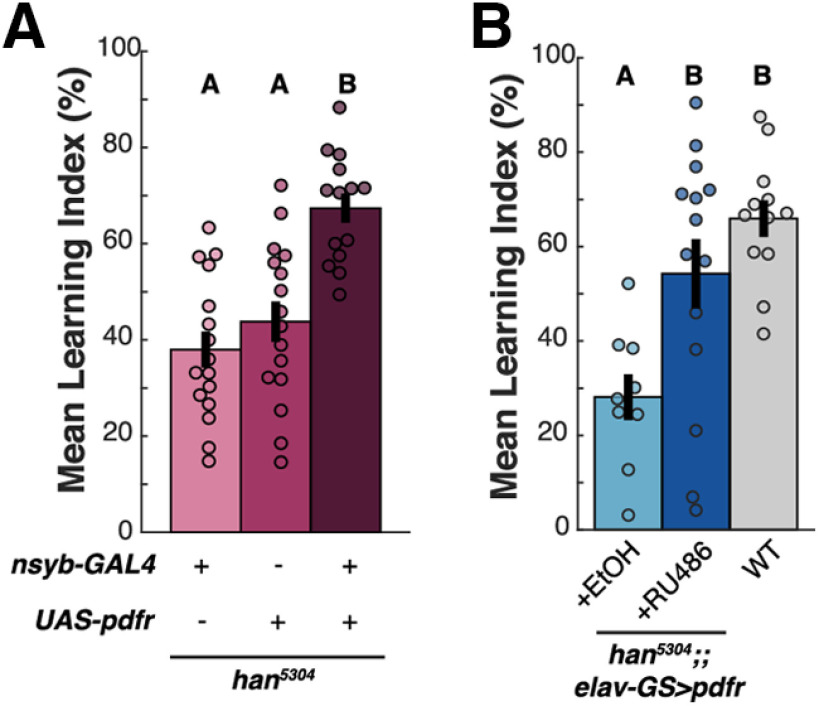
Adult-specific neuronal PDF-PDFR signaling is sufficient for appetitive STM formation. Appetitive olfactory STM tested between ZT0–ZT4 in *han^5304^* mutants with panneuronal PDFR expression. ***A***, Constitutive PDFR expression, compared with genetic controls, tested at ZT0–ZT2 and ***B*** adult-specific PDFR expression using the RU486-inducible GeneSwitch system, compared with EtOH vehicle and WT controls, tested at ZT0–ZT4. Mean LI scores are shown ± SEM, with individual datapoints (circles). Letter categorization indicates groups of statistical similarity (*p* > 0.05) or difference found by one-way ANOVA with Bonferroni *post hoc* comparisons (all significant comparisons, ***A***: *p* < 0.0005, ***B***: *p* < 0.05).

In the mature adult fly, PDF is uniquely produced in the LNv neurons of the clock, but PDFR is present in both neurons and glia (Im and Taghert, 2010). To distinguish between a neuronal and a glial PDF target involved in STM, we asked whether neuron-specific expression of a *Pdfr* cDNA would rescue the *han^5304^* appetitive learning deficit. Panneuronal expression of *Pdfr* on a *han^5304^* background using the *nsyb-GAL4* driver significantly increased STM compared with parental controls at ZT0–ZT2 (one-way ANOVA, *p* = 4.61E-06; *post hoc* comparisons *p*_gal4,UAS_ = 0.808, *p*_gal4,UAS+gal4_ = 6.10E-06, *p*_UAS,UAS+gal4_ = 2.07E-04; Fig. 2*A*). We excluded the possibility of neomorphic effects from the broad *nsyb-GAL4* expression pattern by investigating the same panneuronal PDFR overexpression on a WT background, which failed to generate significant changes in STM compared with parental controls (data not shown). Therefore, we concluded that PDF/PDFR signaling in neurons is sufficient for normal appetitive STM.

To determine whether adult-specific PDF signaling was sufficient for normal STM, we used the drug-inducible driver *elav-GS-GAL4* to panneuronally express *Pdfr* on the *han^5304^* mutant background solely during adulthood. Flies were placed onto food containing RU486 or EtOH vehicle directly after eclosion to fully induce transgene expression (Osterwalder et al., 2001; Depetris-Chauvin et al., 2011). Under these conditions, *han^5304^;;elav-GS>Pdfr* flies fed with RU486 and tested at ZT0–ZT4 (Fig. 2*B*) showed significantly increased appetitive STM compared with their vehicle-fed sibling controls and had learning scores equivalent to the concurrently run WT control (one-way ANOVA, *p* = 8.0235e-04; *post hoc* comparisons *p*_EtOH,RU486_ = 0.0144, *p*_EtOH,WT_ = 5.872e-04, *p*_RU486,WT_ = 0.3288). Taken together, we conclude that PDFR in a population of adult neurons promotes appetitive STM.

### Mushroom body (MB) Kenyon cells (KCs) are not direct targets of PDF

**Figure 3.**
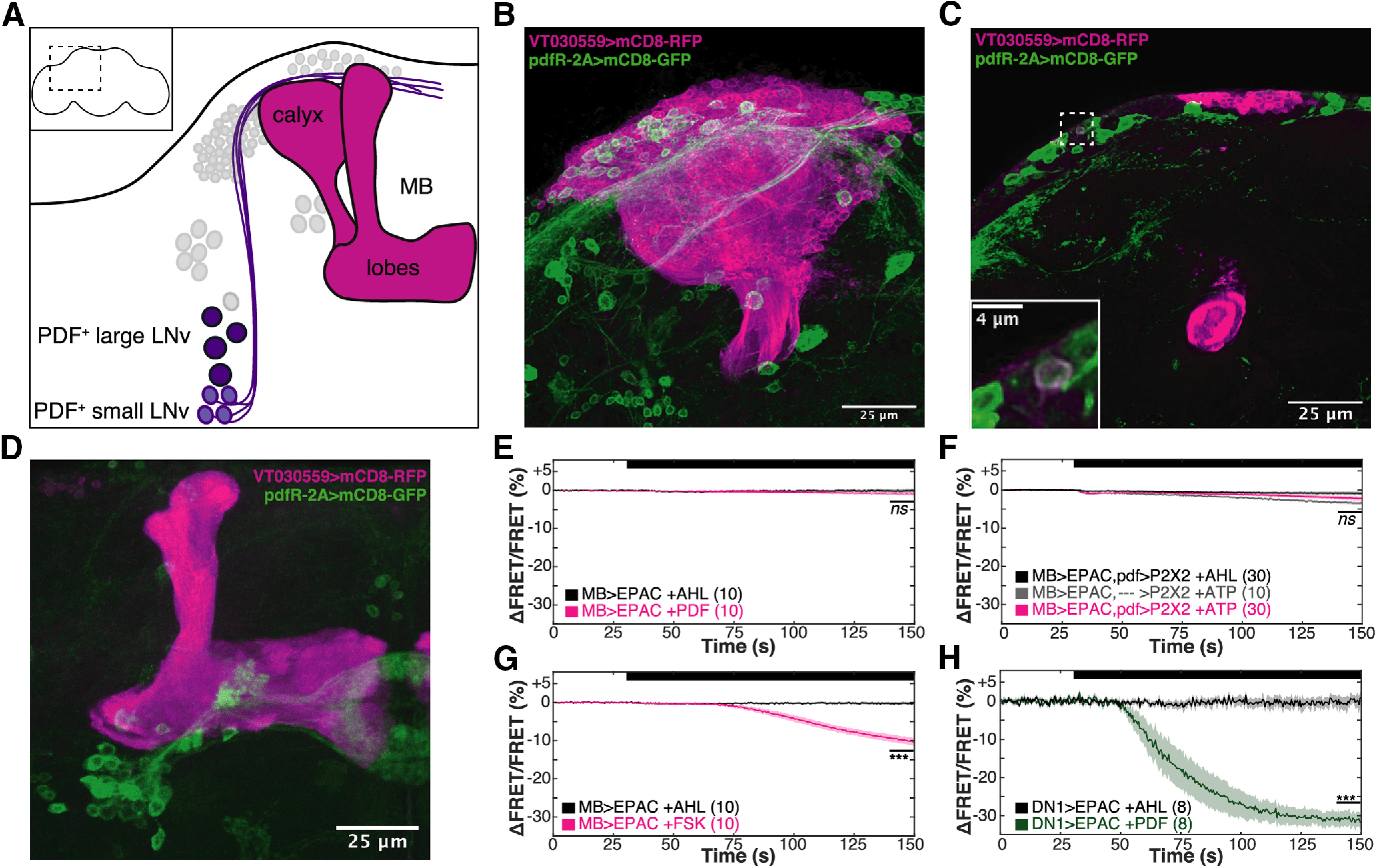
The MB is not a direct target of the PDF signaling pathway. ***A***, Cartoon representation of LNv/MB anatomy. Purple PDF^+^ LNv soma (light: small, dark: large) are shown with other core clock cells in light gray. sLNvs send projections anterodorsomedially in close proximity to the MB (magenta) calyx, as indicated. Inset, Area shown in main panel indicated by dotted outline on whole brain. ***B–D***, Confocal images of MB (magenta) and putative *Pdfr* (green) expression patterns. ***B***, Maximum intensity projection (MIP) of calyx, with single planar slice shown in ***C*** where dotted outline shows the only cell with colocalization of markers seen in *n* = 3 brains (magnified, inset). ***D***, MIP of MB lobe region. ***E–H***, Data shows MB calyx or clock neuron DN1 intracellular cAMP as reported by EPAC1camps FRET signal imaged at 2 Hz (using *MB-LexA* or *clk4.1-GAL4*). After a 30-s baseline AHL perfusion, perfusion of drug (ATP, PDF, FSK) or AHL vehicle was bath applied for the remainder of the trial, as indicated by the black bar atop each panel. The mean signal of the terminal 10 s was used for statistical comparisons. Line mean, shadow ± SEM, *N* of each condition shown in parentheses. ***E***, Calyx EPAC response to bath-applied 30 μm PDF, compared with AHL vehicle control, performed at ZT2–ZT6. Paired *t* test comparison is non-significant. ***F***, LNv neurons were activated by *pdf-GAL4* expression of the ATP-stimulated P2X2 channel and calyx EPAC responses were compared among AHL vehicle and empty driver controls. Data were pooled from five timepoints spaced throughout the 24-h cycle; *t* test comparisons are non-significant. ***G***, Calyx EPAC response to bath-applied 15 μm forskolin (FSK) compared with AHL vehicle control, performed at ZT2–ZT6. Paired *t* test, *p* = 6.71E-06. ***H***, Clock neuron DN1 somatic response to bath-applied 30 μm PDF compared with AHL vehicle control, performed at ZT2–ZT6. Paired *t* test, *p* = 3.09E-07.

We began our search for the relevant PDFR^+^ neurons in the MB, a site of integration essential for olfactory learning and memory in the fly. Olfactory information is delivered to the MB neuropil by sparse random activation of subpopulations of roughly 2000 KCs whose soma surround the calyx of the MB. Within the compartmentalized MB neuropil lobes, KC axonal projections receive stimulus-specific information from dopaminergic inputs, allowing for time-space coincidence of odor-stimulus pairings (for review, see Busto et al., 2010). Dorsomedial projections from PDF^+^ sLNv clock cells skew posteriorly in close proximity to the MB KCs and calyx (Fig. 3*A*; Helfrich-Förster, 1995); while these projections contain small clear core vesicles (Yasuyama and Meinertzhagen, 2010) the recently published *Drosophila* adult brain connectome shows no direct synaptic routes between sLNv and MB KC neurons (https://neuprint.janelia.org and Xu et al., 2020) permitting us to exclusively focus on sLNv extrasynaptic dense core vesicle signaling as a communication mechanism between sLNvs and MBs. In fact, sLNv projections show TOD-dependent changes in PDF immunoreactivity (Park et al., 2000) and low levels of PDFR mRNA have been detected in some KC subpopulations (Crocker et al., 2016). These prior findings led us to ask whether KCs could be a target of PDF.

We labeled KCs with membrane-bound RFP using the *VT030559-GAL4* driver and looked for colocalization with membrane-bound GFP expressed with *Pdfr-2A-LexA* (Deng et al., 2019), a gene-fusion in the *Pdfr* locus (Fig. 3*B–D*) which should recapitulate the endogenous expression pattern of *Pdfr*. Given the dense packing of the KC soma, we took particular care to maximize our confocal Z-resolution and visualize independent soma by using AiryScan deconvolution. A *z*-axis maximum image projection (MIP) of the KC soma and MB calyx (Fig. 3*B*) shows potential overlap of reporter signal; however, a flythrough of the stacked dataset (Movie 1) shows that the entirety of this “overlap” can be attributed to the extremely close proximity of the PDFR^+^ DN1 clock cell soma and PDFR^+^ DN1/LNv projections to the calyx and KC soma. In an examination of ∼6000 KCs from three independent brains we saw only a single cell that expressed both GFP and RFP (Fig. 3*C*, inset). We also examined the MB lobe region to detect any innervation of the neuropil by PDFR^+^ projections; again, though the *z*-axis MIP implies potential overlap (Fig. 3*D*), examination of 3D rotational data (Movie 2) revealed no detectable PDFR^+^ signal within the MB lobe neuropil. GFP signal closest in proximity belonged to EB-projecting PDFR^+^ cells.

We also attempted to visualize a functional connection between the PDF^+^ LNvs and the MB. PDFR is a G-protein-coupled receptor which activates Gαs to increase intracellular cAMP (Duvall and Taghert, 2012, 2013). To allow us to see changes in cAMP we expressed the FRET-based reporter EPAC1cAMPs (Shafer et al., 2008) in the entirety of the MB with *MB247-LexA*, an approach which we have previously used (Haynes et al., 2015). We investigated the ability of LNvs to directly signal to the MB in two ways. First, we bath applied PDF to MB>EPAC brains (Fig. 3*E*). We failed to detect a significant EPAC response in the MB calyx after bath application of PDF as compared with vehicle controls, tested at ZT2–ZT6 (paired *t* test, *p* = 0.245; Fig. 3*E*). To rule out the need for some LNv-released co-transmitter, we also measured MB cAMP response after activation of LNvs. Activation was accomplished by expressing ATP-gated P2X_2_ channels under the control of *pdf-GAL4* and perfusing dissected brains with ATP (Lima and Miesenböck, 2005; Yao et al., 2012). Although we performed experiments at five different timepoints spanning the 24-h circadian day, we failed to detect any significant response to ATP within the MB calyx or KC soma relative to vehicle or genetic controls (pooled data, Fig. 3*F*; paired *t* test, *MB*>*EPAC,pdf*>*P2X2* ± ATP, *p* = 0.153; unpaired *t* test, *MB*>*EPAC,pdf*>*P2X2*+ATP to genetic controls, *p* = 0.054). Importantly, positive controls with 15 μm forskolin, a direct activator of adenylate cyclase, validate our ability to detect bona fide cAMP signals in MB (paired *t* test compared with vehicle, *p* = 6.71E-06; Fig. 3*G*). We also verified that the same PDF bath treatment induces a significant 30% average EPAC response in DN1 clock neurons, cells known to be PDF responsive (paired *t* test, *p* = 3.09E-07; Fig. 3*H*). The PDFR^+^ neuron(s) involved in the regulation of appetitive STM are thus likely to be extrinsic to the MB, acting as interneurons downstream of the PDF^+^ LNvs to regulate the memory center.

### Intraclock PDF signaling is not sufficient for appetitive STM

The two most extensively characterized functions of PDF are regulation of daily locomotor activity timing and maintenance of clock circuit intracellular cycling (manifest by rhythmic locomotor activity in constant conditions). Both of these functions are conducted by PDF activation of PDFR in neurons of the core clock. Because we found that PDF is required for STM but does not directly signal to memory-relevant MB KCs, we next considered PDF signaling within the clock itself as an indirect regulator of memory formation. If cycling of the core clock was required to produce an STM-promoting non-PDF output or if circadian-regulated locomotion itself was a factor in memory formation, intraclock signaling might be memory relevant. We therefore asked whether PDFR expression sufficient for the rescue of canonical *han^5304^* LD and DD locomotor phenotypes is also sufficient for STM rescue. To do this, we first needed to identify clock-related GAL4 lines that were capable of rescuing these phenotypes.

**Figure 4.**
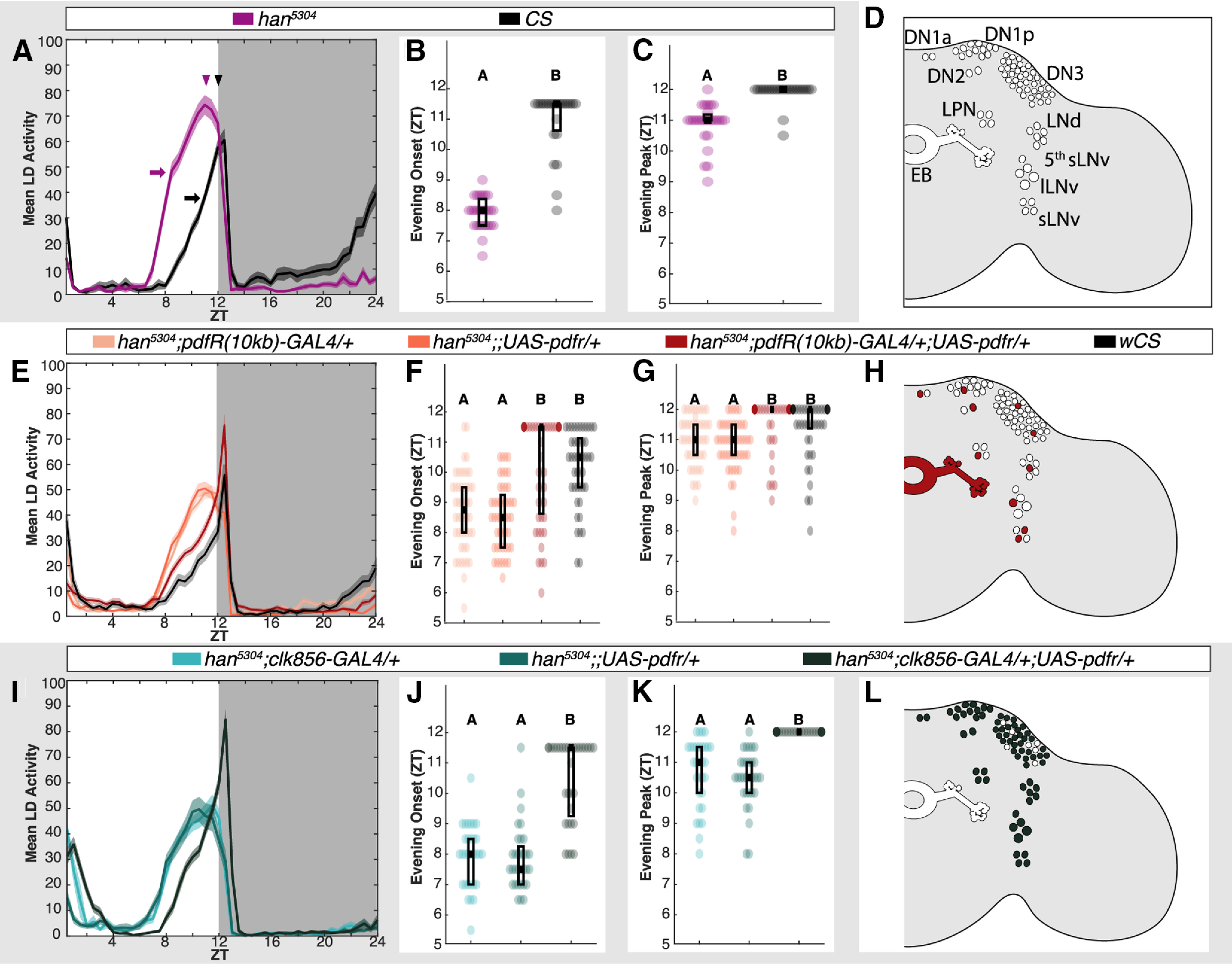
Clock-based PDFR supports normal locomotor activity. LD activity and appetitive STM for *han^5304^* and cell-specific *Pdfr* rescue mutants. ***A***, ***E***, ***I***, Population LD activity (mean ± SEM) shown in 30-min bins averaged from three consecutive days. ***A***, Mean population values of evening anticipation onset (arrows) and evening peak (arrowheads) are shown. Evening anticipation onset (***B***, ***F***, ***J***) and the evening activity peak (***C***, ***G***, ***K***) were extracted for individual subjects (circle datapoints; overlaid box-and-whisker). ***D***, In addition to other cells, core clock neurons (categorized by subtype DN, LNd, LPN, and LNv) and EB soma and ring neuropil in *han^5304^* lack PDFR (indicated by empty outlined ROIs). ***H***, ***L***, Compared with ***D***, filled ROIs demonstrate (***H***) *PdfR(10 kb)-GAL4* and (***L***) *clk856-GAL4* mediated PDFR rescue expression patterns on the *han^5304^* background. Letter categorization indicates groups of statistical similarity (*p* > 0.05) or difference found by (***B***, ***C***) Student's unpaired *t* test (*p* < 1E-7) and (***F***, ***G***, ***J***, ***K***) Kruskal–Wallis with Bonferroni *post hoc* comparisons (all significant comparisons ***F***: *p* < 0.0001; ***G***: *p* < 0.05; ***J***: *p* < 1E-7; ***K***: *p* < 1E-9).

The *han^5304^* LD locomotor activity phenotype has an advance in the timing of evening anticipation onset and an early evening peak activity (Hyun et al., 2005). We observed *han^5304^* activity for three consecutive days in 12/12 LD using the DAM system and, consistent with prior reports, *han^5304^* flies showed accelerated evening onset and evening peak activity compared with WT [Fig. 4*A–C*; Student's unpaired *t* test: *p* = 8.45E-14 (Fig. 4*B*); *p* = 4.38E-08 (Fig. 4*C*)]. We then rescued expression of *Pdfr* in *han^5304^* using two different clock-related drivers: *PdfR(10 kb)-GAL4* and *clk856-GAL4*, and recorded locomotor activity under similar conditions. The *PdfR(10 kb)-GAL4* expression pattern (Fig. 4*H*) includes the ellipsoid body and one to two cells of each clock subset (Parisky et al., 2016). We found that *Pdfr* expression under the *PdfR(10 kb)-GAL4* promotor was sufficient to delay the *han^5304^* evening onset and return evening peak timing back to WT [Fig. 4*E–G*; Kruskal–Wallis with Bonferroni *post hoc* comparisons, *p* = 5.56E-10, *post hoc* comparisons: *p*_gal4,UAS_ = 1.00, *p*_gal4,UAS+gal4_ = 5.44E-05, *p*_UAS,UAS+gal4_ = 2.67E-06, *p*_gal4,WT_ = 3.86E-05, *p*_UAS,WT_ = 1.89E-06, *p*_UAS+gal4,WT_ = 1.00 (Fig. 4*F*); *p* = 9.60E-09, *post hoc* comparisons: *p*_gal4,UAS_ = 1.00, *p*_gal4,UAS+gal4_ = 2.86E-05, *p*_UAS,UAS+gal4_ = 9.71E-08, *p*_gal4,WT_ = 2.67E-02, *p*_UAS,WT_ = 5.61E-04, *p*_UAS+gal4,WT_ = 0.568 (Fig. 4*G*)]. Similarly, clock-limited PDFR expression with *clk856-GAL4* (Fig. 4*L*; Gummadova et al., 2009) significantly delayed the accelerated *han^5304^* evening onset and evening peak timing [Fig. 4*I–K*; Kruskal–Wallis with Bonferroni *post hoc* comparisons, *p* = 1.58E-10, *post hoc* comparisons: *p*_gal4,UAS_ = 1.00, *p*_gal4,UAS+gal4_ = 1.24E-07, *p*_UAS,UAS+gal4_ = 3.40E-09 (Fig. 4*J*); *p* = 1.94E-13, *post hoc* comparisons: *p*_gal4,UAS_ = 0.772, *p*_gal4,UAS+gal4_ = 8.39E-09, *p*_UAS,UAS+gal4_ = 2.96E-12 (Fig. 4*K*)].

The other phenotype of the *han^5304^* mutant is an inability to maintain circadian rhythmicity of locomotor activity after transfer to total darkness (Hyun et al., 2005). Although our STM assays use flies entrained to a 12/12 LD cycle, it was still possible that a PDFR^+^ component of the clock involved in maintenance of circadian rhythmicity was required for STM. We therefore examined the free-running activity of those flies in DD conditions, analyzing activity from DD days 2–8 (Table 2). Compared with WT, *han^5304^* had significantly decreased RI (Student's unpaired *t* test, *p* = 2.84E-08) and periodicity (Welch's *t* test, *p* = 0.0363) and the overall population was much more arrhythmic (70.4% vs 95.6%). PDFR expression with the *pdfR(10 kb)* promotor failed to increase *han^5304^* RI (one-way ANOVA, *p* = 4.75E-14, *post hoc* comparisons *p*_gal4,UAS_ = 0.19, *p*_gal4,UAS+gal4_ = 0.0280, *p*_UAS,UAS+gal4_ = 1, *p*_gal4,WT_ = 4.38E-14, *p*_UAS,WT_ = 1.05E-08, *p*_UAS+gal4,WT_ = 5.575E-07), periodicity (Kruskal–Wallis, *p* = 2.31E-05, *post hoc* comparisons *p*_gal4,UAS_ = 1, *p*_gal4,UAS+gal4_ = 1, *p*_UAS,UAS+gal4_ = 1, *p*_gal4,WT_ = 1.79E-04, *p*_UAS,WT_ = 9.60E-05, *p*_UAS+gal4,WT_ = 6.23E-03) or percent rhythmic values to WT levels. *han^5304^*;*clk856*>*Pdfr* flies had significantly increased RI (one-way ANOVA, *p* = 1.61E-08, *post hoc* comparisons *p*_gal4,UAS_ = 0.013, *p*_gal4,UAS+gal4_ = 8.37E-04, *p*_UAS,UAS+gal4_ = 8.16E-09) and percent rhythmic population values compared with their parental controls, while periodicity showed decreased variance but no rescue in mean values (Kruskal–Wallis, *p* = 1.49E-03, *post hoc* comparisons *p*_gal4,UAS_ = 0.263, *p*_gal4,UAS+gal4_ = 9.27E-04, *p*_UAS,UAS+gal4_ = 0.219).

**Table 2.** Free-running circadian locomotor activity properties of *han^5304^* mutants and rescue genotypes

Analysis group	Genotype	RI	% Rhythmic	Periodicity
**1**	**han^5304^**	**0.267 ± 0.026**	**70.4**	**23.5 ± 0.281**
	**WT**	**0.501 ± 0.021**	**95.6**	**24.1 ± 0.047**
2	han^5304^;pdfR(10 kb)-GAL4/+	0.218 ± 0.017	50.0	23.5 ± 0.233
	han^5304^;;UAS-Pdfr/+	0.275 ± 0.016	72.9	23.3 ± 0.137
	han^5304^;pdfR(10 kb)-GAL4/+;UAS-Pdfr/+	0.295 ± 0.021	73.9	23.6 ± 0.155
	WT	0.445 ± 0.020	93.5	24.0 ± 0.071
**3**	**han^5304^;clk856-GAL4/+**	**0.308 ± 0.025**	**71.0**	**23.1 ± 0.154**
	**han^5304^;;UAS-Pdfr/+**	**0.216 ± 0.019**	**60.7**	**23.8 ± 0.293**
	**han^5304^;clk856-GAL4/+;UAS-Pdfr/+**	**0.422 ± 0.019**	**93.8**	**23.7 ± 0.055**

Flies used in Figure 4 were released into free-running DD conditions for 8 d following Figure 4 LD recordings; circadian properties are calculated from DD days 2–8 and reported as mean ±SEM. Bold analysis groupings indicate independent experimental comparison groups. Statistical differences (*p* < 0.05) were found as follows. Analysis group 1 (RI: Student's unpaired *t* test, *p* = 2.84E-08; periodicity: Welch's *t* test, *p* = 0.0363); analysis group 2 (RI: one-way ANOVA, Bonferroni *post hoc* comparisons *p*_gal4,UAS+gal4_ = 0.0280, *p*_gal4,WT_ = 4.38E-14, *p*_UAS,WT_ = 1.05E-08, *p*_UAS+gal4,WT_ = 5.575E-07; periodicity: Kruskal–Wallis *post hoc* comparisons *p*_gal4,WT_ = 1.79E-04, *p*_UAS,WT_ = 9.60E-05, *p*_UAS+gal4,WT_ = 6.23E-03); analysis group 3 (RI: one-way ANOVA, *post hoc* comparisons *p*_gal4,UAS_ = 0.013, *p*_gal4,UAS+gal4_ = 8.37E-04, *p*_UAS,UAS+gal4_ = 8.16E-09).

**Figure 5.**
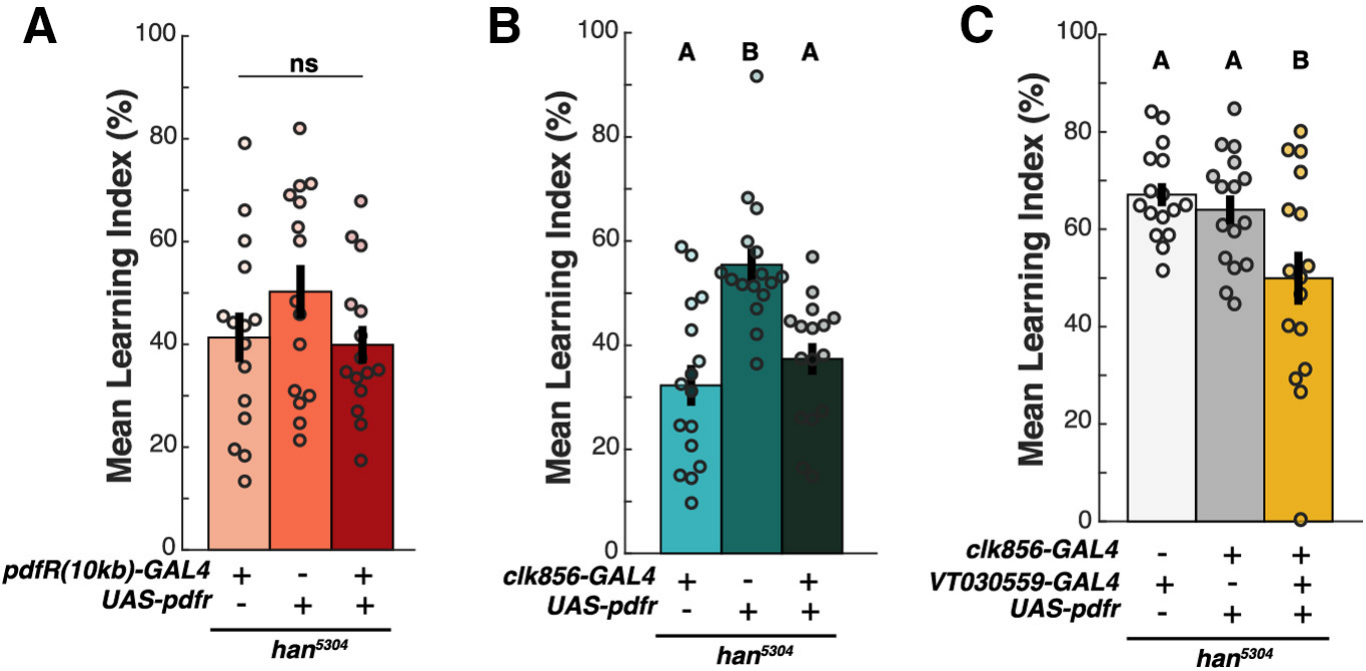
PDF signaling in the clock and MB does not support STM. Appetitive STM of *han^5304^* mutants with cell-specific constitutive *Pdfr* expression. ***A–C***, Mean LI scores are shown ± SEM, with individual datapoints (circles). Letter categorization within each panel indicates groups of statistical similarity (*p* > 0.05) or difference found by one-way ANOVA with Bonferroni *post hoc* comparisons (all significant comparisons, ***B***: *p* < 0.005; ***C***: *p* < 0.05). Flies were tested at (***A***) ZT3–ZT5, (***B***) ZT3–ZT10, and (***C***) ZT1–ZT7.

Taken together, we find that *pdfR(10 kb)-GAL4* driven expression of PDFR rescues only LD activity phenotypes of *han^5304^* mutants, while *clk856-GAL4* PDFR expression is sufficient to rescue both LD and DD phenotypes. These drivers therefore allowed us to ask whether PDF's role in appetitive STM is mediated through core-clock-driven LD locomotor patterns or maintenance of circadian rhythmicity. Both of these hypotheses were disproven as we found that neither *pdfR(10 kb)-GAL4* (tested at ZT4) nor *clk856-GAL4* expression of PDFR (tested at ZT3–ZT10) was able to rescue the *han^5304^* appetitive STM deficit [one-way ANOVA, *p* = 0.239 (Fig. 5*A*), *p* = 3.21E-05 (Fig. 5*B*), *post hoc* comparisons: *p*_gal4,UAS_ = 4.19E-05, *p*_gal4,UAS+gal4_ = 0.879, *p*_UAS,UAS+gal4_ = 0.001]. Furthermore, as the *clk856-GAL4* expression pattern includes nearly all core clock neurons, it is reasonable to entirely infer that clock-based PDFR expression alone is insufficient to support normal appetitive STM. However, to fully exclude any possible synergistic interaction of PDF with the clock and MB in STM behavior, we co-expressed PDFR in the clock and MB simultaneously using *clk856-GAL4* and the strong KC-specific driver *VT030559-GAL4* on a *han^5304^* background (Fig. 5*C*). Tested at ZT1–ZT7, this manipulation not only failed to rescue appetitive STM compared with parental controls, but in fact further impaired learning, likely because of either genetic background effects or to a neomorphic effect of strong overexpression of PDFR in the MB (one-way ANOVA, *p* = 6.15E-03, *post hoc* comparisons: *p*_VTgal4,UAS_ = 1.00, *p*_VTgal4,clkgal4UAS+VTgal4_ = 8.17E-03, *p*_clkgal4UAS,clkgal4UAS+VTgal4_ = 0.0379). These data imply that PDF must be acting on a PDFR^+^ neuronal target exclusive from both the core clock and the MB KCs to regulate appetitive STM. Our results also argue that the multiple behavioral roles of PDF signaling (on locomotor activity, DD rhythms, and memory) are independently controlled by the expression of PDFR in distinct PDF target neurons.

### Aversive olfactory STM requires PDF but not PDFR

Differential distribution of PDFR, the only known receptor for PDF, within and external to the clock allow it to have many different and independent behavioral roles. Other signaling pathways, however, often use an additional strategy for diversification: multiple receptors. We were struck by the difference in STM scores between *pdf^01^* and *han^5304^* mutants (Fig. 1*B*), the *pdf^01^* STM phenotype is significantly and consistently more severe than *han^5304^*. Since both of these alleles are protein null (Hyun et al., 2005; Renn et al., 1999) in theory they should have the same magnitude of deficit if PDFR is the sole receptor for PDF. If, however, there were a second receptor for PDF, it could provide some PDF signaling in the *han^5304^* mutant and result in a milder phenotype.

**Figure 6.**
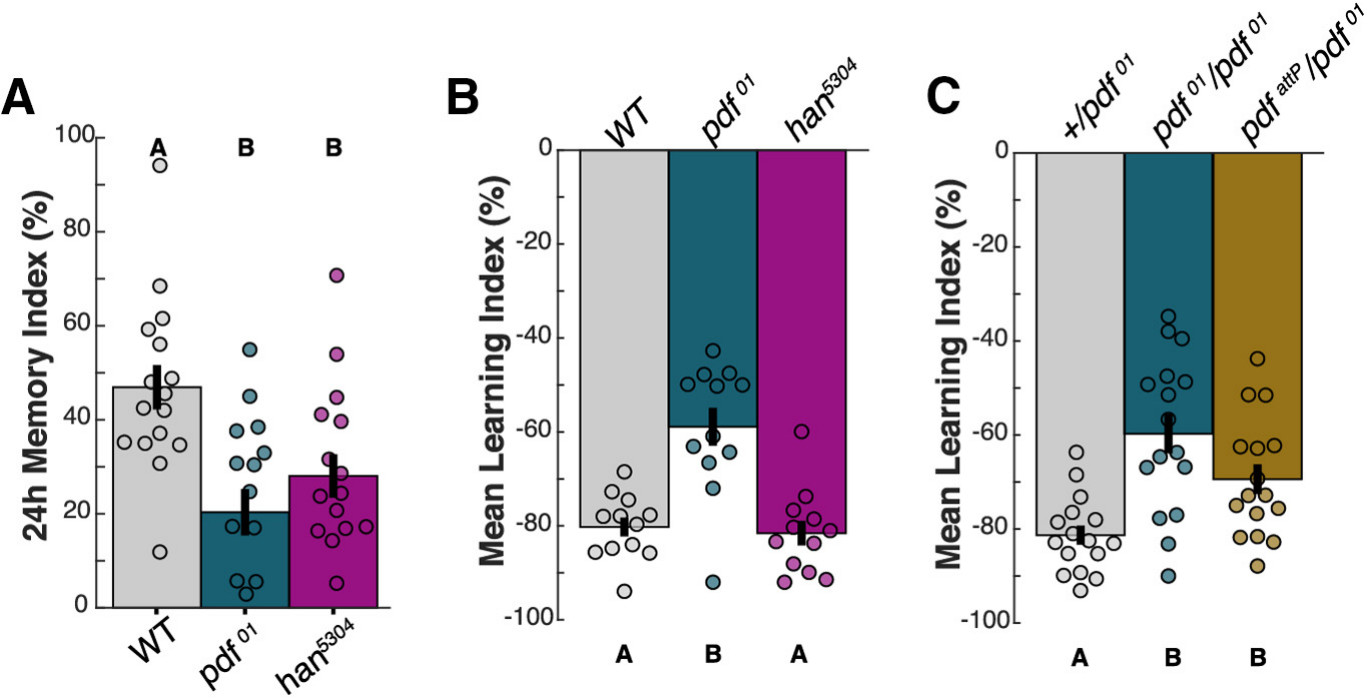
Multiple receptor targets permit valence-specific support of STM by a single clock output. Mean LI scores are shown ± SEM, with individual datapoints (circles). ***A***, Twenty-four-hour appetitive LTM of *pdf^01^* and *han^5304^* mutants. ***B***, ***C***, Aversive STM of WT and PDF pathway mutants. Letter categorizations indicate groups of statistical similarity (*p* > 0.05) or difference found by one-way ANOVA with Bonferroni *post hoc* comparisons (all significant comparisons, ***A***: *p* < 0.05; ***B***: *p* < 1E-4; ***C***: *p* < 0.05). Flies were tested at (***A***) ZT2–ZT5 (***B***) ZT0–ZT2, and (***C***) ZT0–ZT3.

To further explore the learning and memory roles of PDF and PDFR and determine whether the disparity in STM deficits was common to other types of memory, we assayed appetitive LTM and aversive STM. We first investigated a possible difference between *pdf^01^* and *han^5304^* 24 h appetitive LTM, tested between ZT2–ZT4 (Fig. 6*A*). While *pdf^01^* flies were slightly more impaired than *han^5304^* flies were, there was no statistically significant disparity between the mutants (one-way ANOVA, *p* = 8.00E-04, *post hoc* comparisons: *p*_WT,pdf01_ = 7.79E-04, *p*_WT,han5304_ = 0.0215, *p*_pdf01,han5304_ = 0.770). However, we found strong evidence for the existence of a second PDF receptor when we tested *pdf^01^* and *han^5304^* mutants for aversive STM (Fig. 6*B*). In this assay, flies are tested at ZT0–ZT2 for memory to an odor that was previously paired with shock; instead of approaching the paired odor (as in the appetitive assay), flies demonstrate memory by avoiding the paired odor. As before, *pdf^01^* mutants had impaired aversive STM compared with WT, but quite surprisingly, *han^5304^* mutants showed no aversive STM deficit at all (one-way ANOVA, *p* = 5.08E-06, *post hoc* comparisons: *p*_WT,pdf01_ = 4.90E-05, *p*_WT,han5304_ = 1.00, *p*_pdf01,han5304_ = 1.97E-05). To definitively confirm a requirement for PDF in aversive STM and rule out the influence of genetic background in *pdf^01^*, we crossed *pdf^01^* flies to WT, *pdf^01^*, and the recently created *pdf^attP^* mutant (Deng et al., 2019) and tested progeny for aversive STM at ZT0–ZT3 (Fig. 6*C*). *pdf^01^*/+ heterozygotes were indistinguishable from WT for STM, confirming the recessive nature of the defect. *pdf^01^* homozygotes and *pdf^attP^*/*pdf^01^* transheterozygotes had significant STM defects (Kruskal–Wallis, *p* = 4.78E-04, *post hoc* comparisons: *p*_CS/pdf01,pdfattP/pdf01_ = 0.0269, *p*_CS/pdf01,pdf01/pdf01_ = 3.91E-04, *p*_pdfattP/pdf01,pdf01/pdf01_ = 0.676). Collectively, these data suggest that PDF may promote aversive STM by acting *not* on PDFR, but on a novel, and still unidentified, receptor.

## Discussion

**Figure 7.**
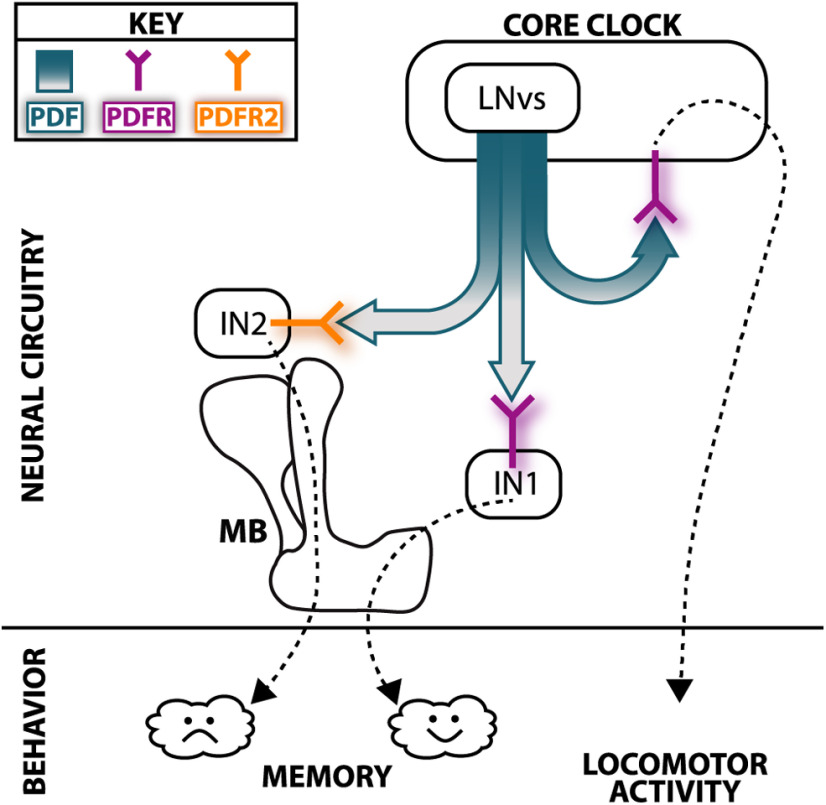
The core clock regulates distinct behaviors via discrete PDF targets: a proposed model. Localization of PDFR to the clock permits control of locomotor activity independently from control of memory. Signaling through PDFR in a population of interneurons extrinsic to both the clock and MB (here shown as IN1) permits regulation of appetitive memory. We propose that, in place of PDF-PDFR signaling, PDF activation of a novel unidentified receptor (here called PDFR2) in a separate population of interneurons (IN2) is required for aversive memory.

A role for the circadian clock in cognition and memory has been demonstrated in many organisms, including humans (for review, see Gerstner and Yin, 2010; Smarr et al., 2014; Krishnan and Lyons, 2015). Efforts to understand these phenomena have largely involved manipulations of intracellular molecular oscillator components [e.g., *period* (*per*)] to abolish intracellular clock cycling. However, this type of manipulation is not fully representative of the nature of human circadian misalignment disorders, as a role for intercellular signaling has also been shown (Yamaguchi et al., 2013). In *Drosophila*, where investigators have tools to interrogate molecular processes in great mechanistic detail, manipulation of the signaling peptide PDF allowed us to examine the role of a key neuromodulatory output of the core clock circuit in cognition. Our work demonstrates a requirement for PDF in the formation of associative olfactory memory (schematized in Fig. 7). Putting our data in the context of what is known about the molecular clock's influence on cognition, we explore below the importance of TOD on WT and PDF mutant STM and suggest the possible existence of a second PDF receptor.

### PDF acts on multiple targets to regulate behaviors

In the adult fly, we and others have shown that PDF released from the LNv neurons of the core clock circuit acts, via PDFR, both on the clock circuit itself and outside of the clock to drive key circadian features and rhythmicity of locomotor activity (Fig. 4; Table 2). Our data show that PDF and PDFR are also required for robust appetitive olfactory memory and for equalizing the ability to form memory across the day (Figs. 1, 2). An additional layer of valence-specific control is provided by the fact that PDF appears to act in aversive olfactory memory formation independently of PDFR, suggesting the possibility of a second receptor (Fig. 6). Surprisingly, intraclock circuit PDF signaling, which is sufficient for normal locomotor activity, plays no role in PDF's regulation of memory, demonstrating that PDF independently directs multiple behaviors depending on the location of its target receptor.

While regulation of associative olfactory memory requires PDF signaling outside both the clock and the MB KCs (Figs. 3–5), the identity of the PDF-responsive element is unknown. Examination of the recently completed hemibrain EM connectome shows no monosynaptic pathways between sLNvs and KCs (https://neuprint.janelia.org; Xu et al., 2020), but a recent study supports the existence of a functional connection since activation of LNvs was sporadically able to produce a GCaMP signal in KCs (Pírez et al., 2019). Given the EM data, this is likely because of sLNv regulation of an excitatory KC input neuron (Fig. 7).

### The role of circadian rhythms themselves in olfactory STM formation is limited

In *Drosophila*, a requirement for molecular clock components like *per* in cognitive tasks has varied, dependent on the type of learning and stage of memory studied (Gailey et al., 1991; Sakai et al., 2004; Lyons and Roman, 2009; Le Glou et al., 2012; Fropf et al., 2014, 2018; Chouhan et al., 2015; Inami et al., 2020). Interestingly, in cases where the locus of action for *per* has been mapped, PER acts outside the core clock circuit to regulate memory, but these studies do not explore TOD effects. In studies examining TOD effects on cognition (Lyons and Roman, 2009; Fropf et al., 2014, 2018), *per* mutants or animals rendered arrhythmic by altered light conditions lose TOD differences. Thus, the role of the cycling core clock itself in learning and memory has been mostly restricted to TOD modulation, and PER, like PDF and PDFR, has an apparent role in memory that is independent of its function in the core clock circuit.

### PDF signaling opposes a latent TOD rhythm in appetitive STM

Our data suggest that the requirement for PDF in supporting normal levels of appetitive STM is TOD-independent i.e., PDF is required at all TODs to form robust appetitive STM. PDF has been implicated in a wide variety of behaviors that are influenced by circadian time (Chen et al., 1999, 2016; Mertens et al., 2005; Chung et al., 2009; Keene et al., 2011; Kim et al., 2013; Krupp et al., 2013; Nagy et al., 2019), but its only previously known roles in learning and memory are courtship related (Inami et al., 2020). The PDF peptide is well-placed to be an agent of general learning enhancement since it can act on long-range targets via extrasynaptic release and diffusion, allowing it to provide neuromodulation over the entire circadian cycle. The fact that PDF has been characterized as an arousal-promoting signal (Parisky et al., 2008; Chung et al., 2009) may be germane to this role since arousal state is critical to attention in working memory (Ricker et al., 2018).

An intriguing feature of the *pdf* and *Pdfr* mutant phenotypes is the emergence of a latent TOD oscillation in STM (Fig. 1). Daily changes in sLNv structure and PDF accumulation at terminals (Park et al., 2000) have maxima and minima at ZT1 and ZT13 (Fernández et al., 2008; Gorostiza et al., 2014) which align with our observed peak and trough of appetitive STM in PDF signaling mutants. The role of PDF in morning arousal (Parisky et al., 2008) may explain the early day appetitive STM trough of *pdf* and *Pdfr* animals: since a heightened arousal state facilitates learning, an absence of PDF signaling reduces morning arousal and cripples the ability of animals to make associations with food-predictive odors. In the absence of measurements of the kinetics of learning, however, it is difficult to pinpoint which stage of the learning process is defective. But the latent nature of TOD effects in mutants relative to the stable nature of WT STM throughout the day allows us to infer that this stability is the sum of oscillating contributions from PDF signaling and one or more other cycling factors. In humans, multiple oscillators for working memory have been proposed (Folkard et al., 1983).

### TOD-independent appetitive memory has survival value

The diversity of roles for PDF in behavior illustrates how essential the output of the clock is for optimal function and survival. But why, in the context of appetitive STM (which is ultimately TOD-independent), is an organism's capacity for appetitive learning so critically linked to its timekeeping capabilities? Although behaviorally-diverse prior work may, on some levels, appear conflicting regarding TOD influence on associative STM (Lyons and Roman, 2009; Fropf et al., 2018), TOD effects are consistently found in associative LTM (Lyons and Roman, 2009; Fropf et al., 2014, 2018; Chouhan et al., 2015, 2017). The need for stability of appetitive STM may be because of the fact that at the time of training, an organism lacks information necessary to evaluate the benefit of devoting metabolic resources to consolidation of that learning, i.e., future food availability is unknown. Though the most widely-used appetitive STM paradigm requires starvation for expression of memory immediately after training, significant expression of appetitive LTM requires starvation only at the time of retrieval but not at the time of acquisition (Krashes et al., 2009; Chouhan et al., 2017). Furthermore, feeding after appetitive training prompts the decay of appetitive STM within 10–30 min, while LTM can still be observed whether animals are re-starved (Krashes et al., 2009). In light of a STM requirement for PDF throughout the day, we hypothesize that PDF may help maximize information acquisition at the front end of the process, to allow memory to be dispensed with or consolidated in a manner dependent on future internal metabolic state information.

### PDF signaling in aversive memory may use a novel receptor

PDF is also required for aversive olfactory STM. Surprisingly, however, there is no requirement for PDFR, the only characterized receptor for this peptide. For *pdf* mutants, it is unlikely that aversive STM loss is because of a second site mutation since it appears in transheterozygous animals as well. And though a valence-specific compensatory mutation in the *han^5304^* line could mediate this phenomenon, it is hard to envision a mechanism for this. Aversive associative STM is therefore the only behavior known for which PDF is required but PDFR is dispensable. In other organisms, circadian output peptides have multiple receptors. Three GPCRs for the *C. elegans* homolog of PDF have been identified, each of which is highly similar to *Drosophila* PDFR and related to VIPR1 and VIPR2, the two known mammalian receptors for VIP (a mammalian peptide with similar clock roles; Janssen et al., 2008, 2009). We suggest that receptor diversity allows the valence-specific regulation of associative STM by regionally segregated and distinct receptors. Since the circuitry involved in acquisition of appetitive versus aversive memory involves different sets of neurons (Riemensperger et al., 2005; Liu et al., 2012; Yamazaki et al., 2018), this implies that PDF will act at circuit nodes in each pathway that are valence-specific and is consistent with our finding that PDF does not act on KCs, the final common substrate of memory. Ultimately, identification and characterization of the second PDF receptor will be required to fully understand this peptide's multiple roles in behavior.
